# Gut Microbiota-Driven Pathways Linking Chronic Stress to Tumor Progression

**DOI:** 10.7150/ijbs.119630

**Published:** 2026-01-01

**Authors:** Qing Li, Siyuan Xia, Xian Zhang, Yuqiang Liu, Xue Xiao, Jinlin Yang

**Affiliations:** 1 Department of Gastroenterology and Hepatology, Sichuan University-University of Oxford Huaxi Joint Centre for Gastrointestinal Cancer, Frontiers Science Center for Disease-Related Molecular Network, West China Hospital, Sichuan University, Chengdu, China.; 2 Department of Pathology, Sichuan University-University of Oxford Huaxi Joint Centre for Gastrointestinal Cancer, Frontiers Science Center for Disease-Related Molecular Network, West China Hospital, Sichuan University, Chengdu, China.

**Keywords:** Chronic stress, Tumor progression, Gut microbiota, Dysbiosis.

## Abstract

Chronic stress is increasingly recognized as a critical factor influencing tumor progression, but its underlying mechanisms remain incompletely understood. This review examines the role of gut microbiota as a critical mediator linking chronic stress to tumor progression. Recent evidence suggests that chronic stress triggers gut dysbiosis, characterized by reduced microbial diversity, depletion of beneficial bacteria, and enrichment of potentially harmful species. We summarize the mechanisms by which chronic stress regulates gut microbial dysbiosis, including stress-related hormone signaling, intestinal inflammation, mucosal barrier disruption, and altered gut motility. Additionally, we examine how stress-induced dysbiosis contributes to tumor progression through immune suppression, metabolic reprogramming, enhanced tumor stemness, and potentially through barrier dysfunction, and chronic inflammation. We further discuss potential therapeutic interventions, including specific probiotics, prebiotics and other strategies that may help suppress tumor development by modulating the stress-microbiota-cancer axis. In conclusion, these emerging insights provide a foundation for novel therapeutic strategies that target the stress-microbiome-cancer axis, which may help suppress tumor progression and complement conventional cancer treatments to improve clinical outcomes in cancer patients.

## 1. Introduction

Cancer is a major public health concern and a leading cause of mortality, with an estimated 20 million new cancer diagnoses and approximately 9.7 million cancer-related deaths worldwide in 2022 1. The global cancer burden continues to rise, with increasing incidence and mortality rates in many regions 2. Chronic stress, characterized by the sustained physiological response to emotional pressures, is now considered not only a psychological concern but also a factor that may influence the course of various diseases 3. There is growing evidence that psychological stress is prevalent among cancer patients and is more frequently being considered a risk factor for cancer development and progression 4.

Chronic stress is believed to influence the occurrence, development, recurrence, and metastasis of various types of cancer through alterations in the neuroendocrine system 5-7. Psychological stress can activate the hypothalamic-pituitary-adrenal (HPA) axis and the sympathetic nervous system (SNS), leading to the release of stress hormones such as cortisol, catecholamines (epinephrine and norepinephrine), and other neurotransmitters 8. These stress mediators can directly or indirectly affect cancer cells and their microenvironment, promoting tumor growth 5, 9, angiogenesis 10, 11, metastasis 7, 12, and immune evasion 13-15. From a clinical perspective, pharmacological blockade of these hormonal pathways has yielded mixed results. For instance, a recent study reported no overall association between β-blocker use and breast cancer prognosis, except for a potential benefit in triple-negative breast cancer patients 16. A meta-analysis similarly found that β-blocker use had no significant effect on cancer-specific survival overall, but revealed substantial heterogeneity across cancer types, showing potential benefit in melanoma and breast cancer while even suggesting harm in pancreatic and head-and-neck cancers 17. These findings imply that the cancer-promoting effects of chronic stress may not be fully explained by neurohormonal signaling alone, and that additional mechanisms in this process need further investigation.

The human gut microbiota, a complex ecosystem of trillions of microorganisms, plays a pivotal role in facilitating bidirectional communication along the gut-brain axis through microbial metabolite signaling and neuroimmune modulation 18. Chronic stress has been associated with reduced microbial diversity, increased intestinal permeability, and a shift towards a pro-inflammatory gut microbiome profile 19, 20. Simultaneously, gut microbial dysbiosis is now widely recognized as an important factor promoting tumor development and progression 21, with current research indicating that approximately 20% of cancers are closely associated with microbiome 22. Given these evidences, we may infer that these stress-induced changes in the gut microbiota could contribute to the development and progression of cancer.

Emerging evidence further positions the gut microbiota as a critical mediator of stress-induced effects on tumor biology 23-25. In this review, we aim to dissect the mechanisms by which chronic stress influences the gut microbiota and, in turn, how these changes can modulate tumor progression. By understanding the complex interplay between chronic stress, gut microbiota, and cancer, we hope to identify potential therapeutic targets and guide the development of novel interventions that integrate stress reduction and microbiota manipulation to improve cancer outcomes.

## 2. Epidemiological evidence linking chronic stress and tumor progression

Tumor development is a process involving complex interactions among genetic, environmental, and lifestyle factors 26, 27. Recent epidemiological studies have suggested a close link between chronic stress and tumor progression. A large-scale meta-analysis by Chida *et al*. examined the relationship between psychosocial stress and cancer incidence by pooling data from 142 prospective studies. They revealed that individuals experiencing psychosocial stress had a 6% higher likelihood of being diagnosed with cancer compared to those with lower stress levels (hazard ratio = 1.06, 95% confidence interval 1.02-1.11, *P* = 0.005), providing robust evidence that stress is a risk factor for cancer 28. Further supporting this, Wang *et al*. performed another systematic review and concluded that psychosocial stress was associated with an increased incidence of lung cancer, oral cavity cancer, prostate cancer and skin cancer 29. Moreover, a prospective study involving 3,015 women found that stress levels were significantly associated with the overall cancer risk 30. In addition to the increased risk of cancer incidence, chronic stress has also been shown to negatively affect cancer outcomes. This is evidenced by increased cancer-specific mortality in patients with breast cancers, lung cancer, colorectal cancer, hematopoietic system cancer, prostate cancer, kidney cancer and bladder cancer who experienced psychosocial stress 29. Moreover, emotional distress (ED), a common manifestation of psychosocial stress, has been associated with poorer clinical outcomes in patients with advanced non-small-cell lung cancer treated with immune checkpoint inhibitors (ICIs). Compared to patients without ED, patients with ED exhibited significantly shorter median progression-free survival (7.9 vs. 15.5 months), a lower objective response rate (46.8% vs. 62.1%), and a reduced 2-year overall survival rate (46.5% vs. 64.9%) 31.

Although these studies provide valuable insights into the relationship between chronic stress and tumor progression, a comprehensive review summarizing the findings of previous research is necessary to better understand the mechanisms underlying this association and to identify potential areas for future investigation.

## 3. Chronic stress reshapes the intestinal flora

While previous studies have elucidated various mechanisms by which chronic stress directly promotes tumor progression through neuroendocrine hormones 4, 8, these pathways may not fully explain the relationship between stress and cancer. Growing evidence suggests that the influence of chronic stress extends beyond direct neuroendocrine regulation, involving more complex systemic changes 8. Among these, the gut microbiota, a key component of the gut-brain axis disturbed under stress 32, may serve as an important bridge connecting chronic stress to tumor biology.

### 3.1 Chronic stress leads to disturbance of gut microbiota

It is estimated that the human microbiome comprises around 10^14^ bacterial cells, which is 10 times the number of human cells 33. Among these, the gastrointestinal microbiota accounts for the majority of the total bacterial population in the human body and is predominantly composed of the phyla Bacteroidetes and Firmicutes 34. Under normal conditions, the gut microbiota maintains a dynamic balance between beneficial and potentially pathogenic bacteria. However, chronic stress disrupts this balance, leading to alterations in microbial composition and function that critically mediate the impact of stress on host health.

#### 3.1.1 Chronic stress leads to changes in the composition of gut microbiota

In rodent models, chronic stress exposure, such as chronic restraint or social defeat, consistently reduce microbial diversity, marked by declines in α diversity (the species richness and evenness within a single sample), and increased β diversity (the difference in species composition between samples) 35, 36. These findings are corroborated by a systematic review demonstrating that psychological stress negatively correlated with α diversity indices, including the Shannon, Chao1, and Simpson indices, while being positively associates with β diversity metrics, such as weighted UniFrac distances, further highlight stress-induced structural disruptions in the microbial community 37. Beyond disruptions in overall diversity, chronic stress drives specific changes in microbial taxa in animal models 38-44, typically characterized by the expansion of pro-inflammatory species and the depletion of beneficial commensals. For instance, in the water avoidance stress (WAS) model, mice exhibited a 2-fold decrease in Bacteroidetes and a 2 to 3-fold increase in Firmicutes and Gammaproteobacteria, suggesting a dysbiotic profile linked to pro-inflammatory potential 45. Similarly, in the chronic restraint stress model, mice showed an increase in pro-inflammatory taxa such as Peptostreptococcaceae,* Helicobacter*, *Streptococcus*, and *Enterococcus faecalis* 20. Notably, these shifts are often accompanied by the overgrowth of pathobionts, including increased relative abundance of Muribaculaceae*, Enterorhabdus, Marvinbryantia* and *Candidatus Arthromitus* 19. Concurrently, chronic stress depletes bacteria that are critical for maintaining gut homeostasis. *Lactobacillus*, a genus known for its anti-inflammatory properties, was significantly reduced under chronic stress conditions 46. Metagenomic analyses further highlight stress-associated declines in *Bacteroides* and *Alistipes*, which are involved in bile acid metabolism and inflammation suppression, while increases in Parasutterella and Rikenellaceae_RC9_gut_group correlate with intestinal dysregulation 47. Additionally, chronic stress decreases the abundance of Lachnospiraceae and *Roseburia*, which produce short-chain fatty acids (SCFAs) that are essential for barrier integrity and immune regulation 20. In addition, numerous studies have reported alterations in gut microbiota under different stress models 48-52, which we have summarized in **Table 1**.

These preclinical findings are supported by emerging clinical evidence demonstrating parallel microbial alterations in humans exposed to chronic psychosocial stressors 39, 53. Students experiencing academic stress exhibited reduced fecal lactic acid bacterial levels 54. Similarly, frontline healthcare workers experiencing psychological stress during the COVID-19 pandemic exhibited gut microbiota dysbiosis with continuously decreasing α-diversity. Beneficial bacteria such as *Eubacterium hallii* and *Lachnospiraceae ND3007* were reduced, both belonging to the *Lachnospiraceae* family, which are known butyric acid-producing bacteria 55. Psychiatric cohorts provide additional support, as depressed patients display altered α and β diversity and compositional shifts across multiple taxonomic levels 56-58. Evidence from gastrointestinal disorders provides additional insight into stress-microbiota interactions. In ulcerative colitis (UC), patients with comorbid depression or anxiety had lower fecal microbial richness and diversity, characterized by an overrepresentation of *Lactobacillales*, *Sellimonas*, *Streptococcus*, and *Enterococcus*, and depletion of *Prevotella* and *Lachnospira* 59. Another study further demonstrated that the relative abundances of Enterobacterales and Enterococcaceae were positively correlated with anxiety and depression scores in UC patients 60. In patients with Crohn's disease, those experiencing high levels of stress showed a significant decrease in the phylum Firmicutes and genus *Anaerostipes*, along with a significant increase in *Parabacteroides* 61. These microbial alterations were accompanied by metabolomic dysregulation 59, 61. Longitudinal evidence from the Swiss IBD Cohort Study further identify this association, among 171 participants in clinical remission, higher perceived stress was linked to significantly lower mucosal microbial α diversity, while anxiety and depressive symptoms correlated with β diversity differences. Taxa from Lachnospiraceae, Fusobacteriaceae, Ruminococcaceae, Veillonellaceae, Alcaligenaceae, Desulfovibrionaceae, and Bacteroidaceae were consistently reduced in individuals with higher psychological distress 62.

Together, these findings indicate that chronic stress consistently reduces microbial diversity, depletes benificial taxa, and favors a pro-inflammatory microbial profile, those changes may amplify systemic inflammation and contribute to cancer-promoting immune dysregulation. A summary of stress-associated alterations in gut microbiota across animal models and human studies is provided in **Table 1**.

#### 3.1.2 Chronic stress leads to changes in the metabolic function of gut microbiota

Beyond taxonomic disruption, chronic stress profoundly reprograms microbial metabolic networks. One important effect is on tryptophan metabolism in the microbiota-gut-brain axis, where long-term stress shifts the balance toward the kynurenine metabolic pathway 63. Separately, chronic social defeat in mice decreased the prevalence of pathways associated with the synthesis and metabolism of neurotransmitter precursors and SCFAs 64. Bile acid metabolism represents another pathway affected by chronic stress. A previous study showed that chronic stress can reshape bile acid metabolism by altering the abundance of Ruminococcaceae, a family central to secondary bile acid production, which may lead to elevated levels of hydrophobic secondary bile acids such as deoxycholic acid 65. These compounds emerged as drivers of intestinal barrier dysfunction and pro-tumorigenic signaling 66, 67. Chronic stress also depletes protective microbial metabolites. In murine models, chronic restraint stress exposure significantly reduced the abundance of gut microbial metabolites phosphatidylethanolamine (PE) and hemolytic phosphatidylethanolamine [LysoPE (15:0/0:0)] in mice, and the reduction of these two metabolites supports a link between chronic stress-altered gut microbial metabolites and enhanced colorectal cancer growth and metastasis 68.

Human studies corroborate these findings, demonstrating that stress-associated microbiota alterations (e.g., reduced *Faecalibacterium* and *Bacteroides*, increased *Blautia* and *Collinsella*) correlate with disrupted lipid metabolism 69. Specifically, diminished fecal cysteine levels, a glutathione precursor, compromise antioxidant defenses, exacerbating oxidative damage to the intestinal epithelium 70. Extending beyond these metabolic pathways, another study provided additional insights into stress-related dysbiosis effects. Comparative analyses of depressed and non-depressed subjects revealed 279 differentially expressed bacterial synthetic proteins, primarily involved in glucose and amino acid metabolism 71, highlighting how psychological distress impacts microbial protein expression and consequently impacts host physiological function.

Collectively, chronic stress transforms the gut microbiota into a pro-inflammatory and metabolically dysfunctional state. This dual assault, driven by microbial taxonomic imbalance and metabolic perturbation, erodes intestinal barrier function and sustains systemic inflammation, thereby creating a permissive microenvironment for disease progression.

### 3.2 Mechanisms by which chronic stress promotes dysbiosis of gut microbiota

**Figure 1:**
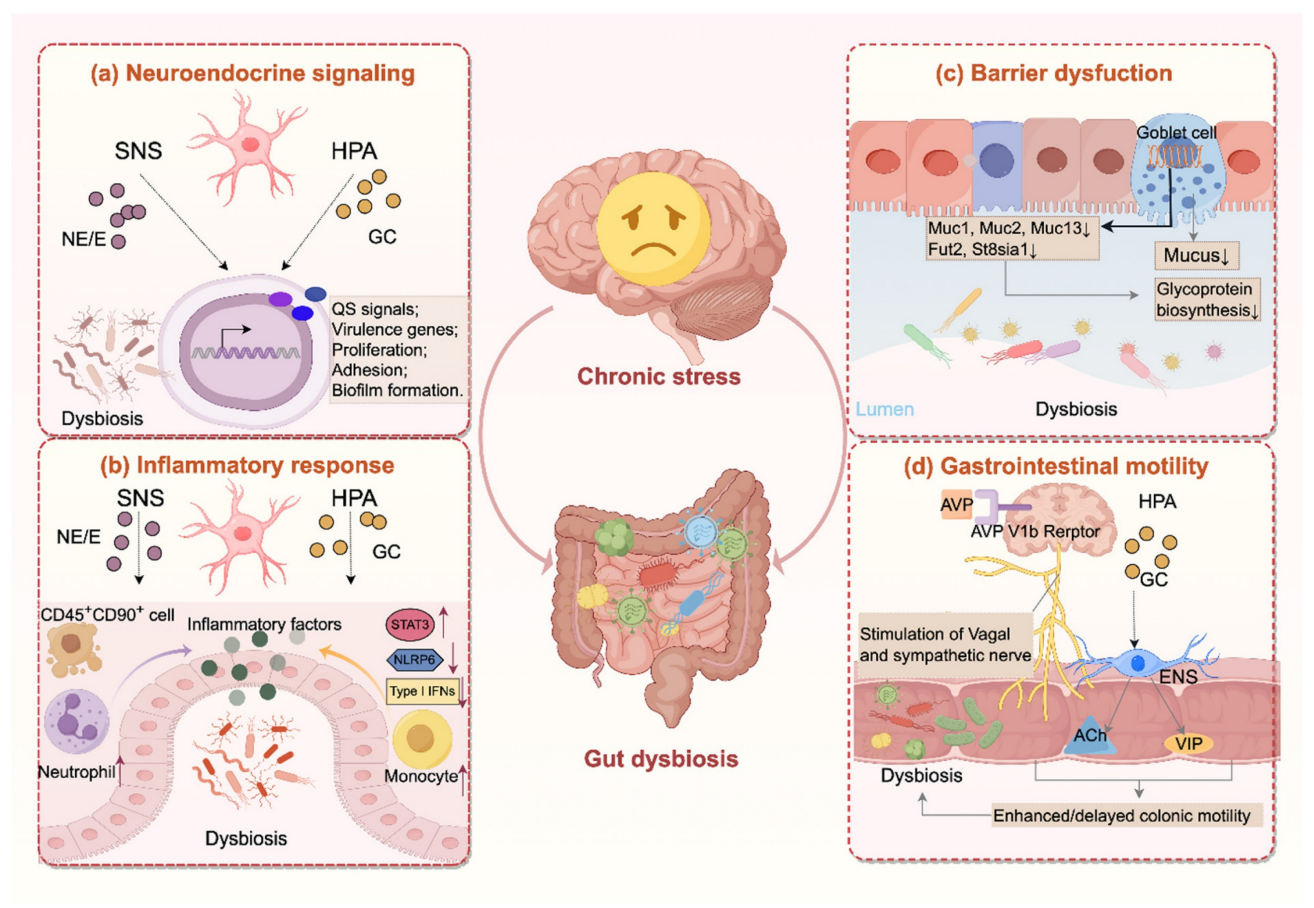
** Chronic stress disrupts the gut microbiota through multiple pathways**. **(a)** Chronic stress activates the hypothalamic-pituitary-adrenal (HPA) axis and the sympathetic nervous system (SNS), elevating glucocorticoids and catecholamines that regulate quorum sensing (QS), bacterial proliferation, adhesion and virulence gene expression, and biofilm formation. **(b)** Hormones released during chronic stress exacerbate intestinal inflammation through multiple mechanisms, including activation of pro-inflammatory signaling pathways (e.g., STAT3), inhibition of type I interferon responses, recruitment of immune cells (e.g., neutrophil, monocyte), and disruption of inflammasome activity (e.g., NLRP6). **(c)** Moreover, chronic stress weakens the mucus barrier by reducing mucin biosynthesis and glycosylation and decreasing goblet cell numbers. **(d)** In addition, chronic stress alters gut motility through AVP V1b receptor-mediated activation of sympathetic and vagal pathways, and glucocorticoid receptor-dependent Ach/VIP release from enteric neurons, leading to both hypermotility or hypomotility that disrupt microbial stability. Together, these changes drive dysbiosis characterized by reduced diversity, depletion of protective taxa, and overgrowth of pro-inflammatory species. Ach, acetylcholine; AVP, arginine vasopressin; E, epinephrine; ENS, enteric nervous system; GC, glucocorticoid; NE, norepinephrine; VIP, vasoactive intestinal peptide.

Chronic stress drives the gut microbiota through multiple mechanisms, involving neuroendocrine signaling, inflammatory response, barrier dysfunction, and alterations in gastrointestinal motility. An overview of these pathways is presented in** Figure 1**.

#### 3.2.1 Chronic stress regulates gut bacteria directly through stress-related hormones

Chronic stress simultaneously activates the HPA axis and the SNS, resulting in sustained release of stress hormones including cortisol and catecholamines. Within the HPA axis, stress stimulation drives the hypothalamus to secrete corticotropin-releasing hormone (CRH), which triggers adrenocorticotropic hormone (ACTH) release from the anterior pituitary and subsequent cortisol secretion from the adrenal cortex 72. Under physiological conditions, excessive cortisol exerts a negative feedback loop that suppresses CRH and ACTH production 73. However, chronic stress disrupts this glucocorticoid-mediated feedback system, resulting in persistently elevated or reduced cortisol levels 74, 75. Meanwhile, sensory information is processed by the prefrontal cortex and transmitted to the amygdala, and the latter relays signals to the dorsomedial hypothalamus (DMH) via the stria terminalis. By sending excitatory signals to brainstem regions including the rostral medullary raphe and rostral ventrolateral medulla, which house sympathetic premotor neurons projecting to the spinal cord, the DMH activates the SNS. These premotor neurons engage sympathetic preganglionic neurons that release acetylcholine, stimulating nicotinic receptors on adrenal medullary chromaffin cells, leading to systemic secretion of epinephrine and norepinephrine 76.

Accumulating evidence suggests that these hormones can directly interact with gut microbiota, modifying their growth and survival, thereby reshaping microbial composition, metabolic function, and pathogenic potential 77, 78. Although direct evidence for the effect of cortisol on intestinal microbiota remains limited, microbial transcriptomic data from other mucosal sites show that cortisol reprograms bacterial gene expression, upregulating genes related to proteolysis, iron acquisition, and motility 79. By contrast, catecholamine-bacteria interactions are well characterized. Physiological plasma concentrations of catecholamines typically range from 23-85 pg/mL for epinephrine and 176-386 pg/mL for norepinephrine, but during or following stress these levels may rise sharply, often by 5 to 20-fold 77. At such stress-relevant concentrations, studies have demonstrated that catecholamines differentially regulate key bacteria. Norepinephrine inhibits *Porphyromonas gingivalis* growth while decreasing quorum-sensing signals, while enhancing virulence-associated rgpB expression 80. Both epinephrine and norepinephrine suppress the growth of *Eikenella corrodens* and* Prevotella intermedia,* yet promote the proliferation of *Fusobacterium nucleatum* and *Tannerella forsythia* 81. Epinephrine further enhances adhesion, biofilm formation, and virulence across a broad range of pathogens, including *Pseudomonas aeruginosa, Enterococcus faecalis, Enterotoxigenic Escherichia, Klebsiella pneumoniae, Staphylococcus aureus* and *Escherichia coli* 82-85. Mechanistically, catecholamines act as interkingdom signaling molecules sensed by bacterial two-component systems such as QseC/QseS, thereby promoting growth, motility, chemotaxis, biofilm formation and virulence gene expression 86. Consistently, *in vivo* studies demonstrate that norepinephrine facilitates bacterial translocation to mesenteric lymph nodes, spleen, and liver 87, highlighting a critical role for stress-induced catecholamines in shaping host-microbe interactions.

Together, these findings suggest that stress hormones release under chronic stress could act as microbial growth signals, directly reshaping gut microbial composition and pathogenic potential.

#### 3.2.2 Chronic stress alters gut microbiota by fostering a pro-inflammatory environment

Mounting evidence indicates that the gut microbiota is altered under inflammatory condition in gut tract 88. Since intestinal inflammation destabilizes commensal communities and selectively promotes the expansion of bacterial taxa with genetic adaptations enabling them to exploit nutrient resources enriched under inflammatory conditions 89. Therefore, as studies have demonstrated that chronic stress establishes an intestinal inflammatory environment, this may contribute to microbial dysbiosis.

At the transcriptional level, stress activates β-adrenergic signaling, linking sympathetic activity to a gene expression program characterized by upregulated pro-inflammatory genes and suppressed type I interferon responses 90. Consistently, elevated CRH under stress suppresses intestinal NLRP6 inflammasome, thereby aggravating intestinal inflammation and reshaping the microbiota 45. Stress hormones also perturb intestinal immune cell homeostasis. Stress-induced glucocorticoid triggers apoptosis of CD45^+^CD90^+^ cells, impairing IL-22-dependent epithelial repair and allowing overgrowth of pathobionts associated with Crohn's disease 91. Elevated catecholamines activate β-adrenergic signaling and subsequently the STAT3 pathway, thereby upregulating pro-inflammatory cytokines (IL-1β, IL-6, IL-17A, IL-22) and neutrophil chemokines (CXCL1, CXCL2), which drive neutrophil infiltration and exacerbate intestinal inflammation 92. The presence of such a systemic low-grade inflammatory state is supported by clinical evidence, as a systematic review of 24 studies reported significantly higher plasma concentrations of TNF-α and IL-6 in patients with major depression 93.

Beyond these pathways, the enteric nervous system (ENS) is another critical mediator of stress-induced inflammatory responses. Persistently elevated glucocorticoid levels generate inflammatory subsets of enteric glial cells (EGCs) that promote monocyte recruitment through CSF1 production and induce inflammation via TNF secretion 94. In the colon tissue of rats under WAS, EGCs also regulate the activity of nitric oxide synthase (NOS) and cholinergic neurons in the ENS, leading to a significant decrease in the abundance of Firmicutes, Proteobacteria, *Lactobacillus*, and Lachnospiraceae_NK4A136, while increasing the relative abundance of Actinobacteria Ruminococcaceae_UCG-005 and Christensenellaceae-R-7 95.

Taken together, these findings suggest that chronic stress induces intestinal inflammation through diverse neuroendocrine and immune mechanisms, and given that intestinal inflammation is a well-established driver of microbial dysbiosis, this may partly explain how chronic stress perturbs gut microbial homeostasis.

#### 3.2.3 Chronic stress disturbed gut microbiota through impairing gut barrier

The intestinal mucus barrier, consisting of a dual-layered system in the colon, serves as a crucial defense against gut microbes, dietary antigens, and other harmful toxins 96, 97. Beyond its protective role, the mucus layer also shapes the gut microbiota by providing O-glycans that serve as attachment sites and nutrient sources that facilitate bacterial colonization and growth 97, 98. Consequently, factors that disrupt mucus integrity not only allow closer contact between microbes and epithelial cells but also create a selective environment that favors pathobiont overgrowth.

Chronic stress has been reported to impair the intestinal mucus barrier, significantly reducing the thickness of the colonic mucus layer, accompanied by increased bacterial penetration into the inner mucus layer 19, 99. Mechanistically, these effects are linked to transcriptional changes in glycoprotein biosynthesis genes (Muc1, Muc13) and mucin glycosylation genes (Fut2, St8sia1), which disrupt normal mucin synthesis and glycosylation 19. In the duodenum, stress suppresses neuronal activity in the central amygdala (CeA), reducing vagal excitability and thereby inhibiting Brunner's gland mucus secretion, ultimately lowering *Lactobacillus* colonization 100. In the colon, stress downregulates Muc2, together with its positive regulator Cdx2, and decreases goblet cell numbers, collectively impairing mucus production 20. Concurrently, Muc13 expression is also reduced, further compromising mucus barrier 101. In addition, chronic stress also alters mucin O-glycosylation, disrupting the biochemical structure and reducing the cohesiveness of the mucus layer, thereby weakening the barrier integrity 102.

The ultimate consequence of these barrier disruptions is increased intestinal permeability in both rodents and humans, resulting in systemic low-grade inflammation due to bacterial translocation 103, 104, and enabling bacteria to translocate to extraintestinal organ systems 105. Collectively, these findings suggest that chronic stress may perturb the gut microbiota, at least in part, through disruption of the mucus barrier.

#### 3.2.4 Chronic stress modulates gastrointestinal motility and thus changes gut microbiota

Gastrointestinal transit time is a major determinant of microbial composition and metabolic activity 106. Individuals with delayed gastrointestinal transit exhibit a distinct microbial profile, with a marked reduction in Bacteroides abundance and an increase in Firmicutes 107. Clinical observations in constipated patients show significant reductions in *Bifidobacterium* and *Bacteroides* abundance 108, coupled with increased relative proportions of methanogenic archaea compared to healthy controls 109. These findings emphasize the role of normal intestinal motility as a critical factor in maintaining microbial homeostasis 110. Studies have demonstrated that chronic stress stimuli can modulate intestinal motility, which may influence the composition and function of gut microbiota. These motility-associated changes in gut microbiota highlight the importance of understanding the specific mechanisms by which chronic stress affects intestinal transit.

Researches reveal that stress exhibits bidirectional effects on gut motility, either accelerating or decelerating intestinal transit. In terms of accelerated colonic motility, chronic psychological stress has been shown to enhance proximal colonic transit through activation of AVP V1b receptors in the brain 111. Similarly, experimental models simulating chronic stress through maternal separation (MS) and CRH administration increase colorectal motility 112, with CRH modulating colonic activity by regulating both vagal and sympathetic components of the autonomic nervous system 113. Glucocorticoid receptor signaling pathways also contribute to stress-induced enhancement of colonic motility 114. Additionally, rats subjected to chronic unpredictable mild stress (CUMS) exhibit increased numbers of enteric neurons (particularly cholinergic and VIP-secreting motor neurons) and glial cells in the ileal submucosal plexus, further accelerating intestinal transit 115. Conversely, stress can also delay intestinal transit. Clinical evidence indicates that adverse life experiences can influence neurophysiological pathways, exacerbating constipation symptoms 116. A large cross-sectional study confirmed that anxiety states significantly correlate with increased constipation risk (OR: 1.49) 117. Animal studies provide further support, demonstrating that CUMS stimulation prolongs gastrointestinal transit time in mice 118, 119. These stress-induced alterations in gut motility, whether accelerating or slowing transit, modify the intestinal microenvironment by changing factors such as substrate availability in the colon. These changes selectively favor the growth of certain bacterial populations while inhibiting others, ultimately reshaping the composition of the gut microbiome 106.

## 4. Chronic stress promotes tumor progression by regulating gut microbiota

**Figure 2:**
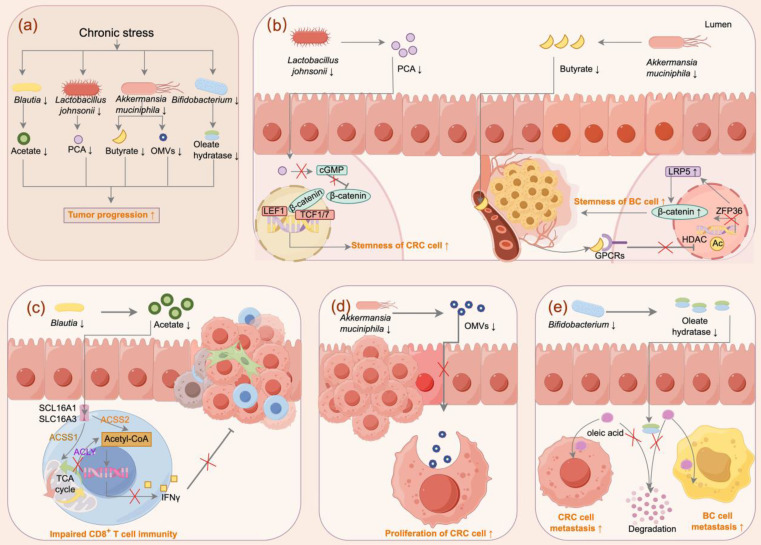
**Microbiota-dependent mechanisms linking chronic stress to tumor progression**. **(a)** Chronic stress depletes beneficial taxa, including *Blautia, Lactobacillus johnsonii, Akkermansia muciniphila, and Bifidobacterium*, leading to reduced microbial metabolites. These changes collectively enhance tumor cell stemness, impair anti-tumor immunity, promote tumor proliferation, and drive metastasis. **(b)** *Lactobacillus johnsonii* depletion reduces protocatechuic acid (PCA), activating Wnt/β-catenin signaling and increasing stemness of colorectal cancer (CRC) cells; loss of *Akkermansia muciniphila* -derived butyrate impairs HDAC inhibition, stabilizes LRP5, and enhances β-catenin signaling, promoting breast cancer (BC) cell stemness. **(c)** *Blautia* depletion reduces acetate levels, impairing acetyl-CoA metabolism in CD8⁺ T cells, thereby suppressing IFN-γ production and anti-tumor cytotoxicity. **(d)** Loss of *Akkermansia muciniphila* decreases outer membrane vesicles (OMVs) release, facilitating CRC cell proliferation. **(e)** *Bifidobacterium* depletion disrupts oleate hydratase activity, downregulated degradation of oleic acid, which enhances CRC and BC metastasis.

Chronic stress profoundly reshapes gut microbiota composition and function, raising the critical question of whether these microbial changes could influence tumor development and progression. Although most mechanistic evidence derives from rodent models, which raises important translational limitations, emerging data directly suggest that gut microbiota and their metabolites can regulate tumor progression by impairing anti-tumor immune surveillance, enhancing tumor cell stemness, and driving the accumulation of pro-tumorigenic metabolites (**Figure 2**).

One important example involves stress-sensitive commensals that sustain CD8⁺ T cell immunity. *Blautia* species are consistently reduced under chronic stress in both breast cancer patients and mouse model, leading to diminished production of acetate, a SCFA essential for CD8⁺ T cell effector function. Supplementation of acetate fuels acetyl-CoA metabolism within CD8⁺ T cells, thereby sustaining IFNγ production and anti-tumor cytotoxicity. Thus, stress-induced depletion of *Blautia* suppresses this pathway and facilitates breast cancer progression 23. Notably, *Blautia* abundance also correlates with improved response to PD-1 blockade, where *Blautia* enhanced CD8⁺ T cell infiltration and suppressed tumor growth 120, 121. *Lactobacillus johnsonii* is another key bacterium found to be depleted in mouse models of colorectal cancer under conditions of chronic stress 24. Restoration of *L. johnsonii* or its metabolite protocatechuic acid (PCA) suppresses β-catenin signaling and inhibits tumor stemness 24. The protective role of *Lactobacillus johnsonii* extends to other cancer types as well. In papillary thyroid carcinoma (PTC), decreased levels of *Lactobacillus johnsonii* are observed in tumor tissues of patients with lymph node metastasis, while supplementation with *Lactobacillus johnsonii* has been shown to inhibit tumor progression by suppressing the Wnt/β-catenin pathway 122. Beyond its direct impact on tumor cell signaling, *Lactobacillus johnsonii* is critical for orchestrating a robust anti-tumor immune response. A fasting-mimicking diet, which increases *Lactobacillus johnsonii* levels, also boosts the infiltration of CD8^+^ T cells into tumors, leading to tumor inhibition 123. Mechanistically, phospholipids from* Lactobacillus johnsonii* can mature bone marrow-derived dendritic cells by upregulating genes related to maturation and migration 124, and it also promotes the synthesis of the metabolite indole-3-propionic acid (IPA), which enhances the efficacy of immune checkpoint blockade (ICB) in multiple tumor types by promoting the differentiation of progenitor exhausted CD8^+^ T cells 125. Another critical commensal, *Akkermansia muciniphila,* is also depleted under chronic stress in both patients and mouse model. In breast cancer, loss of *Akkermansia muciniphila*-derived butyrate, a histone deacetylase (HDAC) inhibitor that suppresses Wnt/β-catenin signaling via LRP5 destabilization, promotes tumor stemness and accelerates tumor progression 25. In colorectal cancer, stress-induced depletion of *Akkermansia muciniphila* impairs the release of protective outer membrane vesicles (OMVs), thereby facilitating tumor cell proliferation 126. Moreover,* Akkermansia muciniphila* has also been recognized as a beneficial commensal with tumor-suppressive functions in colorectal cancer by inhibiting AhR/β-catenin signaling 127. In addition, outer membrane proteins of *Akkermansia muciniphila* such as Amuc_1100 and Amuc_2172 remodel the tumor microenvironment and promote CD8⁺ T cell immunity 128, 129.

Beyond the depletion of protective taxa, chronic stress also reshapes microbial metabolic network. Elevated glucocorticoid levels during chronic stress inhibit *Bifidobacterium* growth, impairing its ability to degrade oleic acid. The resulting accumulation of serum oleic acid enhances metastatic potential in both breast and colorectal cancer 130. Chronic stress also alters bile acid metabolism, marked by increased deconjugation and secondary bile acid synthesis in mice 65. These changes may be mediated in part by bile salt hydrolase (BSH), an enzyme broadly distributed among gut microbes 131, which regulates bile acid pools. Recent work has highlighted the structural diversity of BSH and its pivotal role in modulating host-microbiota interactions, with emerging implications in cancer progression 132. In particular, BSH activity in Bacteroides increases unconjugated bile acids, which activate bile acid receptors and β-catenin/CCL28 signaling, driving Treg-mediated immunosuppression and colorectal cancer progression 67. Together, these findings suggest that stress-induced depletion of protective commensals removes key microbial defenses against tumor progression across cancer types.

In addition to the evidence described above, certain stress-induced alterations may also contribute to the interactions among chronic stress, the gut microbiota, and tumor progression. First, chronic stress promotes intestinal inflammation in a microbiota-dependent manner. Across multiple stress paradigms, stress exacerbates colitis, elevates colonic cytokines and ROS, and induces dysbiosis 133, 134, these effects were reversed in germ-free or broad-spectrum antibiotic-treated mice 135. Given the well-established link between chronic inflammation and tumorigenesis 136, 137, stress-driven pro-inflammatory microbiota may create a permissive milieu for cancer initiation and progression. Second, stress impairs epithelial barriers. In addition to the mucus barrier, the epithelial tight junctions, another critical component of the gut barrier 138, have also been shown to be disrupted under chronic stress, including occludin, TJP1, and TJP2, thereby increasing intestinal permeability and bacterial translocation 139, 140. Mechanistically, CRH enhances paracellular and transcellular permeability 141, promotes autophagy and Paneth cell metaplasia 142. Glucocorticoid signaling is another key contributor, as it reduces the expression of tight junction protein such as occludin both *in vitro* and *in vivo*, effects that can be reversed by the corticosteroid receptor antagonist RU-486 143, 144. This indicates that the changes occur through elevated corticosteroid levels, and likely via activation of epithelial cell glucocorticoid receptors (GR) 145. Mast cell activation further contributes to barrier disruptions, as chronic stress induced mast cell hyperplasia and activation 146, and human data further confirm stress-induced intestinal hyperpermeability via CRH-mast cell pathways 147. Such barrier dysfunction allows pathogenic bacterial translocation and systemic dissemination of microbial metabolites, which may in turn facilitate tumor development in colon and distant organs 148-150. Third, chronic stress diminishes beneficial taxa such as *Lactobacillus* and *Bifidobacterium* 151, which a normally exert anti-inflammatory, antioxidant, and barrier-protective functions 152, 153. For example, S-layer proteins from *Lactobacillus crispatus* interact with DC-SIGN on dendritic cells to attenuate mucosal inflammation in the lower female reproductive 154, while the secreted factor p40 from *Lactobacillus rhamnosus* GG promotes colonic Tregs differentiation and preserves epithelial barrier function under inflammatory stress 155. The enzymatic protein LPH from *Lactobacillus* further contributes to intestinal protection by generating muramyl dipeptide to activate NOD2 signaling 156. Moreover, extracellular vesicles from *Lactobacillus paracasei* (LpEVs) counteract LPS-induced inflammation by reducing the expression of pro-inflammatory cytokine (IL-1α, IL-1β, IL-2, and TNFα) while upregulating IL-10 and TGFβ 157. Certain metabolites of *Lactobacillus* also have direct tumor-suppressive roles, γ-linolenic acid from *Lactobacillus plantarum* MM89 induce ferroptosis in colorectal cancer cells 158, reuterin from *Lactobacillus reuteri* disrupts redox homeostasis to inhibit tumor growth 159, and indole derivatives such as IAA, ICA, ILA, and IPA from *Lactobacillus* modulate Treg differentiation, enhance dendritic cell function, and reprogram CD8⁺ T cell chromatin accessibility to strengthen antitumor immunity and improve responses to immunotherapy 125, 160-162. Moreover, in hepatocellular carcinoma, acetate produced by* Lactobacillus reuteri* suppresses IL-17A release from ILC3s by modulating histone acetylation of Sox13 at site K30, thereby inhibiting tumor growth 163. Their depletion under stress removes critical immunoregulatory pathways, potentially compromising tumor surveillance. Supporting clinical evidence comes from a randomized trial in bladder cancer patients, where oral *Lactobacillus casei* supplementation significantly reduced recurrence rates 164.

In summary, chronic stress appears to promote tumor progression through microbiota-dependent pathways, including immune suppression, metabolic reprogramming, enhanced tumor stemness, barrier dysfunction, and chronic inflammation. Importantly, these microbiota-dependent effects are not uniform across cancers as discussed above. For instance, *Blautia* depletion under stress has been linked to impaired CD8⁺ T-cell immunity in breast cancer 23, whereas another study reported that *Akkermansia muciniphila* constrains stemness in breast cancer 25. In colorectal cancer under chronic stress, loss of *Lactobacillus johnsonii* has been shown to promote stemness 24, while other evidence suggests that *Akkermansia muciniphila* suppresses tumor cell proliferation 126. These discrepancies likely reflect heterogeneity in baseline microbiota across mice, tumor models, stress paradigms and duration, and cancer types.

Moreover, although accumulating evidence suggests that chronic stress, gut microbiota alterations, and cancer progression are linked, most available data remain associative and preclinical. From a causal inference perspective, temporality is insufficiently established because most human studies are cross-sectional. Evidence for a dose-response relationship is lacking, as no studies have systematically compared different levels or durations of stress. By contrast, mechanistic plausibility is relatively well supported by animal studies demonstrating that stress-sensitive taxa and their metabolites can modulate immune surveillance, barrier integrity, and tumor biology. Current human evidence for the stress-microbiota-cancer axis is largely indirect, consisting of studies that examine stress-associated microbial alterations or associations between specific microbial taxa and cancer risk, while research directly connecting all three components remains limited. Nevertheless, several studies have reported associations between stress and microbiota changes, or between microbiota profiles and cancer outcomes, which are summarized in **Table 2.** These findings broadly align with mechanisms established in preclinical models and provide supportive, although not yet causal, evidence that stress-induced microbial dysbiosis may contribute to tumorigenesis. Notably, evidence appears relatively more robust for colorectal and breast cancers, where stress-induced microbial changes and tumor-promoting effects have been repeatedly documented, whereas data for other cancer types remain sparse. Future research should include well-designed studies that vary stress exposures, incorporate temporal assessments, and control for baseline microbiota to strengthen causal inference. Expanding clinical evidence through prospective cohorts and intervention trials will also be essential to validate preclinical findings and define cancer-type-specific pathways, thereby advancing translational opportunities within the stress-microbiota-cancer axis.

## 5. The interplay of confounding factors modulating the stress-microbiota-cancer axis

The pathway linking chronic stress to tumor progression via microbiota disruption does not act in isolation. Instead, it is embedded within a complex network of confounding variables, including diet, host genetics, medication use, and lifestyle, all of which can substantially modulate each component of the axis. Disentangling the specific contribution of stress from these factors is a critical challenge.

### 5.1 Diet

Among all environmental influences, diet is perhaps the most powerful modulator of gut microbiota composition and function 165. Western-style diets rich in saturated fats and emulsifiers promote dysbiosis, reduce SCFAs-producing bacteria such as *Faecalibacterium prausnitzii,* and increase pathobionts, thereby impairing barrier integrity and sustaining low-grade inflammation that can prime tumorigenesis 166, 167. Conversely, diets rich in fiber and polyphenols, such as the Mediterranean diet, foster resilient microbial communities that produce anti-inflammatory metabolites like butyrate, which directly inhibit cancer cell growth and enhance the efficacy of cancer therapies 167. Diet also shapes the stress response itself, since diets rich in fiber, omega-3 fatty acids, vitamin D, and iron are associated with reduced psychiatric symptoms (depression, anxiety, and stress), while poor nutritional status may exacerbate stress-induced pathophysiology 168, 169. Thus, acting as a pivotal link among chronic stress, the gut microbiome, and tumor development, diet plays a dual role in either mitigating or potentiating the adverse consequences of stress. Importantly, dietary background must be considered as a key confounding factor, since it can obscure whether observed microbiota changes are stress-driven or diet-driven.

### 5.2 Host genetics

Host genetics provides a fundamental framework that influences both microbial communities and host responses to chronic stress. Genome-wide association studies have identified specific host genetic variations associated with the abundance of particular microbial taxa 170. By regulating mucosal immunity pathways and epithelial barrier related genes, host genetics establishes the baseline for microbial stability 171, 172. Moreover, genetic variation also affects how individuals respond to stress. Polymorphisms in genes can regulate the HPA axis and neurotransmitter systems, which further contribute to variability in stress reactivity and downstream neuroimmune signaling 173, thereby modifying the extent to which chronic stress perturbs the gut microbiota. Collectively, these mechanisms may help explain the observed heterogeneity in microbiota composition, stress resilience, and cancer outcomes across individuals. Host genetics therefore represents a potential confounding factor in the stress-microbiota-cancer axis, as genetic variability may obscure or amplify the effects attributed to chronic stress. Future studies should integrate host genomics with longitudinal microbiome and metabolome profiling to clarify causal relationships.

### 5.3 Medication

Pharmacological agents are major contributors to microbiota perturbation. Even transient antibiotic exposure can cause rapid diversity loss and long-term shifts, eliminating microbial taxa that are important for immune homeostasis and stress resilience 174, 175. Such dysbiosis has been linked to reduced efficacy of cancer immunotherapy, as the microbiota is essential for priming anti-tumor immune responses 176. While antibiotics provide the clearest example, other commonly used drugs such as proton pump inhibitors (PPIs), antidiabetics (metformin), nonsteroidal anti-inflammatory drugs (NSAIDs) and atypical antipsychotics (AAPs), have also been associated with changes in microbiome composition 177. Antidepressants alter microbial composition as well, raising the possibility that part of their therapeutic effect may be mediated through the gut-brain axis 178. Taken together, these findings highlight the importance of obtaining a comprehensive medication history, particularly regarding antibiotic use, for accurately interpreting microbiome data in clinical research and patient management.

### 5.4 Lifestyle factors

Lifestyle factors strongly influence both microbiota and stress responses. Regular physical exercise enhances microbial diversity, increases SCFA producers, and reduces systemic inflammation, thereby mitigating both stress and cancer risk 179. Conversely, sleep disruption and circadian misalignment induce dysbiosis, hyperactivate the HPA axis, and sustain a pro-tumorigenic inflammatory milieu 180. Furthermore, individuals under chronic stress often adopt unhealthy behaviors such as smoking and excessive alcohol consumption 181, 182. These habits are recognized risk factors for cancer, and they also contribute to microbial dysbiosis and increased intestinal permeability 183-185, thereby amplifying the downstream effects of stress on host physiology. Interactions among stress, poor sleep, and unhealthy habits create feedback loops that synergistically drive tumor development.

Collectively, diet, genetics, medication, and lifestyle are not merely background noise but active participants in the stress-gut microbiota-tumor axis. Failure to account for these confounder risks overestimating the direct effects of stress in human studies. Future investigations must employ rigorous longitudinal designs, incorporate detailed covariate tracking, and apply advanced statistical approaches to disentangle causality. A deeper understanding of these interactions will be essential for developing personalized interventions that target this complex network to improve cancer outcomes.

## 6. Potential therapeutic strategies targeting the stress-microbiota-cancer axis

**Figure 3:**
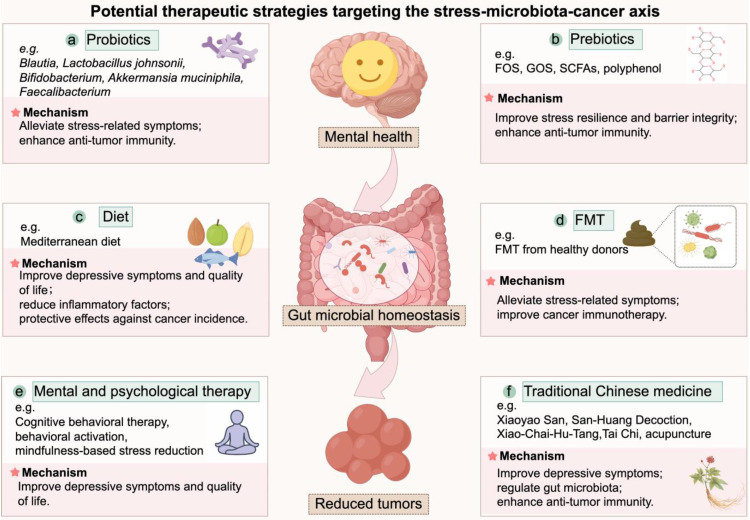
**Potential therapeutic strategies targeting the stress-microbiota-cancer axis**. This schematic summarizes six categories of interventions that modulate the stress-microbiota-cancer axis. **(a)** Probiotics alleviate stress-related symptoms and enhance anti-tumor immunity. **(b)** Prebiotics promote beneficial taxa, improve stress resilience, reinforce barrier integrity, and enhance anti-tumor immunity. **(c)** Dietary interventions improve mental health, reduce inflammation, and lower cancer risk. **(d)** FMT alleviates stress-related symptoms and improves responses to cancer immunotherapy. **(e)** Mental and psychological therapies relieve chronic stress and improve depressive symptoms and quality of life of cancer patients. **(f)** TCMs alleviate stress and modulate gut microbiota and immune function, thereby contributing to anti-tumor effects. FOS, fructooligosaccharides; GOS, galactooligosaccharides; FMT, Fecal microbiota transplantation; SCFA, short-chain fatty acid; TCM, traditional Chinese medicine.

Based on the evidence outlined above, it can be inferred that interventions targeting gut microbiota may serve as a potential strategy to mitigate tumor-promoting effects of chronic stress. Consistent with recent comprehensive reviews, microbiota-directed strategies including probiotics, prebiotics, dietary interventions, and fecal microbiota transplantation (FMT) can reshape the tumor microenvironment and improve therapeutic outcomes in cancers 186, 187. Meanwhile, mental and psychological therapies and traditional Chinese medicine (TCM)-based approaches may improve patient outcomes through the alleviation of chronic stress and related systemic effects. These strategies warrant further investigation in the context of stress-associated cancer progression, an overview of these potential therapeutic strategies was illustrated in **Figure 3**.

### 6.1 Probiotics

As discussed earlier, commensals such as *Blautia, Lactobacillus johnsonii, Bifidobacterium,* and *Akkermansia muciniphila* are consistently depleted under chronic stress, and supplementation in animal models restores immune surveillance and suppresses tumor growth 23-25, 130. In addition to these direct antitumor effects reported above, numerous animal studies report that probiotics alleviate stress-related behavioral and physiological alterations. For instance, pretreatment with *Bifidobacterium adolescentis* reduced anxiety- and depression-like behaviors induced by chronic restraint stress by remodeling the gut microbiota, suppressing hippocampal inflammation, and upregulating brain-derived neurotrophic factor (BDNF) 188. Similarly, *Bifidobacterium breve* CCFM1025 prevents stress-induced emotional disturbances and gastrointestinal dysmotility by restoring microbial balance 189. *Faecalibacterium prausnitzii* also prevented anxiety- and depression-like behaviors in CUMS models and protected against sleep deprivation-induced intestinal injury 190, 191. Importantly, these genera (e.g., *Lactobacillus, Bifidobacterium, Faecalibacterium*) or their metabolites also display anti-tumor activities in preclinical models. *Lactobacillus* species act mainly through metabolite-driven pathways, including reuterin-mediated redox disruption, indole derivatives that enhance T cell immunity, and SCFA-mediated suppression of Wnt/β-catenin signaling 125, 158-163. *Bifidobacterium* species modulate the tumor microenvironment by boosting host immunity, for example, promoting CD8⁺ T cell responses, activating macrophage or recruiting dendritic cells 192-194. *Faecalibacterium prausnitzii* suppresses tumor growth by attenuating inflammation and increasing CD8⁺ T cell infiltration 195. Thus, probiotics may exert dual benefits by alleviating stress-induced dysfunction and directly modulating tumor biology.

Evidence from human studies provides partial but encouraging support. Most probiotic formulations tested in clinical trials for depression are based on *Lactobacillus* and *Bifidobacterium* species 196*.* In one randomized controlled trial, a multi-strain probiotic containing *Lactobacillus* and *Bifidobacterium* significantly improved mood after four weeks of intervention in individuals with depressive symptoms 197*.* Likewise, a meta-analysis of seven randomized controlled trials confirmed that probiotics, particularly *Lactobacillus* and *Bifidobacterium* species, reduced subjective stress levels and alleviated stress-related anxiety and depression in humans 198. However, not all studies reported beneficial effects. For example, an eight-week intervention with *Lactobacillus helveticus* and *Bifidobacterium longum* failed to improve mood in individuals with depressive symptoms 199. These discrepancies suggest that probiotic efficacy may depend on microbial strain, dosage, and intervention duration, and host factors. Supporting this, a meta-analysis concluded that interventions longer than eight weeks and exceeding 10 × 10^9^ CFU were more effective at reducing depressive symptoms 200. Notably, clinical studies have also provided evidence that probiotics can modulate tumor outcomes. In a randomized trial of non-muscle-invasive bladder cancer, oral supplementation with *Lactobacillus casei* significantly reduced tumor recurrence 164. In addition, observational studies in cancer patients receiving immunotherapy have shown that the presence of *Bifidobacterium*,* Lactobacillus* is associated with improved responses to PD-1/PD-L1 blockade 201, consistent with their immunostimulatory roles described in preclinical studies 202, 203. Clinical evidence further indicates that perioperative probiotic supplementation may enhance postoperative recovery in cancer patients by reducing infection rates, alleviating intestinal inflammation, and promoting bowel function 187.

Despite these promising results, several key challenges must be addressed before clinical implementation. As highlighted by Karam et al., although emerging data support the efficacy of probiotic interventions, a deeper understanding of strain-specific functional activities and mechanisms is required before clinical use 204. Moreover, the ability of orally administered probiotics to stably colonize the gut and retain functional activity remains uncertain, as survival is influenced by lysozyme, gastric acidity, pancreatic and bile juice, and colonization resistance from resident microbiota 205-208. Safety concerns must also be considered, especially in immunocompromised or elderly cancer patients, as rare cases of probiotic-associated sepsis have been reported, and horizontal transfer of antibiotic resistance genes to commensal or opportunistic pathogens has been documented 209, 210. The above findings highlight the need for cautious use, strain-level safety profiling, and additional clinical monitoring in vulnerable populations 211. Finally, translational gaps persist due to differences in microbiota composition and host immunity between animal models and humans 212, 213.

These preclinical and clinical findings suggest that targeted probiotic supplementation represents a promising strategy to mitigate the impact of chronic stress and remodel the tumor microenvironment, thereby counteracting chronic stress-driven tumor promotion. However, before it can be incorporated into clinical management, well-designed clinical trials are needed to clarify optimal strains, dosing regimens, and treatment duration, particularly in the context of stress-associated cancer progression.

### 6.2 Prebiotics

In addition to probiotic-based interventions, prebiotics represent another promising strategy, exerting their effects by selectively stimulating the growth or activity of beneficial microorganisms. The International Scientific Association for Probiotics and Prebiotics (ISAPP) recently redefined prebiotics as “a substrate that is selectively fermented by gut microbes, thereby enhancing host health” 214.

Among various candidates, fructooligo-saccharides (FOS) and galactooligosaccharides (GOS) are the most extensively studied in the context of stress and depression 215. In animal models, GOS alone or combination with FOS suppressed stress-induced corticosterone release, alters hippocampal and hypothalamic gene expression, and increased beneficial SCFAs such as acetate and propionate while reducing isobutyrate 216. Other prebiotics such as plant-derived polysaccharides also exhibit anxiolytic and antidepressant effects 217. Moreover, synbiotic formulations (probiotics combined with polyphenol-rich prebiotics) have been shown to mitigate ileal and prefrontal cortical inflammation and ameliorate depressive- and anxiety-like behaviors in mouse models by generating metabolites such as 4-hydroxyphenylpropionic acid and caffeic acid 218. Beyond stress regulation, prebiotics also provide anti-tumor benefits. In mouse models, oral administration of prebiotics enhanced microbial production of SCFAs, thereby suppressing tumor growth and improving drug sensitivity 219, 220. Similarly, inulin supplementation enriched *Bifidobacterium*, increased γδ T cell infiltration in tumors, and inhibited tumor progression 221.

Evidence from human studies, though heterogeneous, is generally favorable. A randomized controlled trial reported that GOS supplementation tended to reduce trait anxiety and improve reaction times in young females with high baseline anxiety 222, while another study showed that GOS supple-mentation alleviated stress-related gastrointestinal dysfunction 223. Recent meta-analyses further concluded that GOS interventions generally lower anxiety levels, though effect sizes vary across populations 224. With respect to cancer, however, clinical data on prebiotics remain limited. Most evidence comes from preclinical models, and large-scale clinical trials with cancer-related endpoints are still lacking 225.

When considering prebiotics for potential clinical translation, both dose and safety profile must be taken into account. Previous study indicated that daily intake of ≥ 5 g of FOS or GOS can improve anxiety and depressive symptoms 168. Besides, prebiotics are generally well tolerated, though higher doses may cause transient, dose-dependent gastrointestinal effects such as bloating, flatulence, and osmotic diarrhea 226. Careful monitoring may be required when prebiotics are used in vulnerable populations, such as elderly individuals or patients with gastrointestinal disorders.

Collectively, these findings indicate that prebiotics exert beneficial effects on stress responses and show promise in suppressing tumor progression. However, their clinical translation remains limited.

### 6.3 Dietary interventions

Dietary interventions are powerful modulators of gut microbiota composition and function 227. In a 20-year prospective cohort of 49,261 Swedish women, higher adherence to a Mediterranean diet in midlife was linked to a significantly lower risk of depression 228. A randomized controlled trial in young men with moderate to severe depression similarly found that a 12-week Mediterranean diet intervention improved depressive symptoms and quality of life 229. Individuals with higher compliance to the Mediterranean diet showed significant reductions in inflammatory markers, including C-reactive protein, TNF-α, and IL-6, which may contribute to gastric cancer prevention, however, the magnitude of this effect varied depending on dietary adherence 230. A recent meta-analysis by Giordano et al. further suggested that adherence to a Mediterranean diet may protect against cancer incidence in older adults 231. These results suggest that dietary modification may help counteract stress-related immune and metabolic dysregulation relevant to cancer progression.

### 6.4 Fecal microbiota transplantation

FMT also provides a direct method to restore microbial balance and has been reported to alleviate stress-related symptoms such as anxiety and depression in patients with irritable bowel syndrome 232, 233. In cancer therapy, two landmark trials showed that FMT restored responsiveness to PD-1 blockade in subsets of melanoma patients 234, 235, highlighting its therapeutic potential in cancer. Although generally considered a safe procedure, FMT may still cause adverse events, most commonly abdominal discomfort, bloating, nausea, and diarrhea, and its potential long-term risks remain insufficiently characterized, warranting further investigation 236. Together, these findings suggest that FMT, by targeting both stress-related dysbiosis and cancer treatment responses, represents a promising yet still experimental strategy within the stress-microbiota-cancer axis.

### 6.5 Mental and psychological therapy

Mental and psychological interventions represent an essential component of comprehensive management for stress-associated cancer progression. Modifying patients' responses to stressors can significantly reduce psychological distress and improve quality of life 237, 238. Among these, cognitive behavioral therapy (CBT) is one of the most extensively studied interventions, with strong evidence supporting its efficacy in alleviating anxiety, depression, and overall psychological distress commonly observed in cancer patients 239. According to the ASCO guidelines, clinicians are recommended to provide CBT, behavioral activation (BA), mindfulness-based stress reduction (MBSR), structured physical exercise, or other empirically supported psychosocial interventions for patients presenting with moderate depressive symptoms 240. A recent review by Anabel *et al*. summarized the impact of stress management interventions on cancer outcomes, highlighting that the effect of psychological therapies on cancer survival remains controversial 4. Some studies have reported improved survival among breast cancer patients receiving psychological interventions 241, 242, whereas others found no significant survival benefit 243, 244. To date, most studies have focused on breast cancer, and the applicability of these findings to other cancer types remains uncertain. Within the stress-microbiota-cancer axis, psychological therapies primarily target the stress response. Their benefits for anxiety, depression, and quality of life are well documented 245, 246. Whether such interventions directly reverse dysbiosis, restore barrier function, or modify tumor-associated inflammation in cancer populations has not been demonstrated and warrants further study. Collectively, psychological therapies may alleviate chronic stress, enhance patients' resilience, and potentially contribute to better clinical outcomes in cancer.

### 6.6 Traditional Chinese medicine (TCM) treatments

According to traditional Chinese medicine (TCM) theory, psychological stress is regarded as a key factor contributing to disease development and progression. Both preclinical and clinical studies have demonstrated its therapeutic potential in alleviating stress-related disorders and inhibiting tumor progression 247, 248. *Xiaoyao San* (XYS), a classical prescription traditionally used to treat mental disorders, was shown to suppress chronic stress-induced hepatic metastasis in a mouse model of colorectal cancer 249. In a clinical study, *San-Huang Decoction* (SHD) alleviated chronic stress caused by long-term endocrine therapy in breast cancer patients, while also inhibiting tumor growth and preventing drug resistance 250. Moreover, Xiao-Chai-Hu-Tang (XCHT) significantly improved depression scores, systemic inflammation, and gut dysbiosis in cancer patients with depressive symptoms. Experimental evidence revealed that XCHT suppressed tumor growth by downregulating the TLR4/MyD88/NF-κB and IL-6/JAK2/STAT3 signaling pathways and improving the tumor immune microenvironment 251, 252. In addition to herbal therapies, mind-body interventions rooted in TCM, such as Tai Chi (TC), resistance training (RT), and traditional Chinese acupuncture (TCA), have shown promising benefits. A randomized controlled trial in elderly cancer patients (aged >55 years) demonstrated that a 12-week TC and RT program improved sleep quality, mental health, and cancer-related fatigue 253. Another clinical study reported that TCA effectively alleviated chronic stress symptoms 254. Collectively, these findings indicate that TCM-based interventions may concurrently modulate psychological, microbial, and immune pathways, providing a systems-level strategy to disrupt the stress-microbiota-cancer axis.

## 7. Conclusion

By recognizing the gut microbiota as a pivotal mediator in the stress-cancer axis, this review provides a framework for developing novel therapeutic strategies that target this axis at multiple levels. Future research should focus on identifying specific microbial signatures associated with stress-induced cancer progression and developing personalized interventions based on individual microbiome profiles.

## Figures and Tables

**Table 1:** Stress-associated alterations in gut microbiota across animal models and human studies.

Category	Model/Population	Main microbiota changes	References
Animal models	Chronic unpredictable mild stress	↑ Bacteroidetes; Bacteroidaceae, Helicobacteraceae, Rikenellaceae, Muribaculaceae.↓ Firmicutes; Eggerthellaceae, Bifidobacteriaceae, Lactobacillaceae, Prevotellaceae, Ruminococcaceae, Lachnospiraceae;* Lactobacillus, Bacteroides, Bifidobacterium.*	40-44
Animal models	Social stress	↑ Parabacteroides; Muribaculaceae;* Enterorhabdus, Clostridium, Flavobacterium.↓ Marvinbryantia, Candidatus Arthromitus, Lactobacillus, Roseburia, Bacteroides, Turicibacter; Lactobacillus johnsoni.*	19, 39, 46, 48, 151
Animal models	Chronic restraint stress	↑ Peptostreptococcaceae; *Helicobacter*, *Streptococcus, Oscillibacter, Gordonibacter; Enterococcus faecalis, Citrobacter rodentium.↓* Porphyromonadaceae; *Parabacteroides, Ruminococcus, Prevotella, Lactobacillus, Alistipes.*	20, 38, 49-51
Animal models	Water avoidance stress	↑ Firmicutes, Gammaproteobacteria, Actinobacteria; Ruminococcaceae, Christensenellaceae-R-7; *Staphylococcus, Erysipelatoclostridium, Streptococcus.*↓ Bacteroidetes, Firmicutes; Proteobacteria, Lachnospiraceae, Muribaculaceae; *Lactobacillus, Bifidobacterium.*	45, 52, 95
Animal models	Emotional stress	↑ *Parasutterella and Rikenellaceae_RC9.*↓ *Bacteroides, Alistipes*.	47
Clinical (psychological stress)	Students under exam stress	↓ *Lactic acid bacteria*.	54
Clinical (psychological stress)	Healthcare workers during COVID-19 experiencing psychological stress	↓ α-diversity; *↓ Eubacterium hallii*, *Lachnospiraceae ND3007.*	55
Clinical (psychological stress)	Patients with depression/anxiety	Altered α- and β-diversity. ↑ Thermoanaerobacteriaceae; *Eggerthella, Holdemania, Gelria, Turicibacter, Paraprevotella, Anaerofilum; Eggerthella lenta, Flavonifractor plautii.*↓Prevotellaceae; *Lactobacillus, Prevotella, Dialister; Ruminococcus bromi, Victivallis vadensis, Ruminococcus bicirculans.*	39, 56-58
Clinical (GI disease + stress comorbidity)	UC patients with depression/anxiety	↓ Richness and diversity.↑Enterobacterales, Enterococcaceae; *Lactobacillales*, *Sellimonas*, *Streptococcus*, *Enterococcus.*↓ *Prevotella*, *Lachnospira*.	59, 60
Clinical (GI disease + stress comorbidity)	CD patients with stress	↑ *Parabacteroides.* ↓Firmicutes; *Anaerostipes.*	61
Clinical (GI disease + stress comorbidity)	Swiss IBD cohort	↓ α-diversity.↓ Lachnospiraceae, Fusobacteriaceae, Ruminococcaceae, Veillonellaceae, Alcaligenaceae, Desulfovibrionaceae, Bacteroidaceae.	62

GI, gastrointestinal; UC, ulcerative colitis; CD, Crohn's disease; IBD, Inflammatory bowel disease.

**Table 2:** Summary of evidence connecting chronic stress, gut microbiota alterations, and cancer progression in animal and human studies.

Microbe / Metabolite	Change under chronic stress	Cancer types	Direct Mechanism in tumor under chronic stress	Other mechanism that related to tumor progression without stress	References
Blautia spp.	↓ (patients, mice)	Breast cancer	↓ Acetate → impaired acetyl-CoA metabolism in CD8⁺ T cells → ↓ IFN-γ and cytotoxicity.	Enhanced CD8⁺ T cell infiltration andimproved better PD-1 response.	23, 120, 121
Lactobacillus johnsonii	↓ (mice)	Colorectal cancer	↓ PCA metabolite → ↑ Wnt/β-catenin → ↑ tumor stemnes.	Suppresses tumor progression by inhibiting the Wnt/β-catenin pathway; Enhances CD8⁺ T cell infiltration into tumors;Promotes DC maturation via bacterial phospholipids; IPA metabolite promotes progenitor exhausted CD8⁺ T cell differentiation and enhancing immunotherapy efficacy.	24, 122-125
Akkermansia muciniphila	↓ (patients, mice)	Breast cancer;Colorectal cancer	↓ Butyrate acts as HDAC inhibitor → ↓ LRP5 destabilization → ↑ Wnt/β-catenin → ↑ tumor stemnes;↓ OMVs → ↑ tumor cell proliferation.	Suppresses Wnt/β-catenin signaling;Outer membrane proteins (Amuc_1100, Amuc_2172) remodel the TME and enhance CD8⁺ T cell immunity.	25, 127-129
Bifidobacterium spp.	↓ (patients, mice)	Colorectal cancer	↓ Oleic acid degradation → ↑ serum oleic acid → ↑ tumor metastasis.	\	130
Bile acid metabolism (BSH activity)	↑ deconjugation and secondary bile acids in mouse models	\	\	Unconjugated bile acids activate bile acid receptors and β-catenin/CCL28 → Treg-mediated immunosuppression.	65, 67, 131, 132
Pro-inflammaroty microbiota	↑ (mice)	\	\	↑ colonic cytokines/ROS, closely related to inflammation associated cancer	133-137

DC, dendritic cell; HDAC, Histone deacetylase; IPA, indole-3-propionic acid; OMVs, outer membrane vesicles; PCA, Protocatechuic acid; TME, tumor microenviroment.

## Abbreviations

ACTH: adrenocorticotropic hormone
BA: behavioral activation
BDNF: Brain-derived neurotrophic factor
CBT: cognitive behavioral therapy
CeA: central amygdala
CRH: Corticotropin-releasing hormone
CUMS: Chronic unpredictable mild stress
ENS: Enteric nervous system
ED: Emotional distress
FOS: Fructooligosaccharides
GABA: γ-aminobutyric acid
GF: Germ-free
GOS: Galactooligosaccharides
HDAC: Histone deacetylase
HNSCC: Head and neck squamous cell carcinoma
HPA: Hypothalamic-pituitary-adrenal
IAA: Indole-3-acetic acid
IBD: Inflammatory bowel disease
ICA: Indole-3-carboxylic acid
ICIs: Immune checkpoint inhibitors
IECs: Intestinal epithelial cells
ILA: Indole-3-lactic acid
IPA: Indole-3-propionic acid
MBSR: mindfulness-based stress reduction
MDD: Major depressive disorder
MS: Maternal separation
NOS: Nitric oxide synthase
PCA: Protocatechuic acid
PE: Phosphatidylethanolamine
ROS: Reactive oxygen species
RT: resistance training
SCFAs: Short-chain fatty acids
XCTH: Xiao-Chai-Hu-Tang
SFB: Segmented filamentous bacteria
SHD: *San-Huang Decoction*
SNS: Sympathetic nervous system
SOC: Social overcrowding
XYS: *Xiaoyao San*
TC: Tai Chi
TCA: traditional Chinese acupuncture
TCM: traditional Chinese medicine
TEER: Transepithelial electrical resistance
WAS: Water avoidance stress
VOE: Vaso-occlusive episodes
4NQO: 4-nitroquinoline-1-oxide
